# Mapping the Evolution of Clinical Trials on Drug-Resistant Tuberculosis: A Bibliometric Overview (1965–2023)

**DOI:** 10.7759/cureus.55190

**Published:** 2024-02-28

**Authors:** Namrata Dagli, Mainul Haque, Santosh Kumar

**Affiliations:** 1 Karnavati Scientific Research Center, Karnavati School of Dentistry, Karnavati University, Gandhinagar, IND; 2 Pharmacology and Therapeutics, National Defence University of Malaysia, Kuala Lumpur, MYS; 3 Department of Periodontology and Implantology, Karnavati School of Dentistry, Karnavati University, Gandhinagar, IND

**Keywords:** clinical trials, overlay visualization, network visualization, network analysis, multidrug-resistant, mycobacterium, tuberculosis, drug-resistant, citation analysis, bibliometric

## Abstract

This study provides a comprehensive overview of the current landscape in drug-resistant tuberculosis research. An extensive electronic search was conducted on PubMed and Scopus databases to identify clinical trials related to drug-resistant tuberculosis. Network analysis and visualization were performed on the data using the Biblioshiny App and VOSviewer software. This bibliometric study focuses on revealing publication trends, leading contributors, key institutions, thematic focuses, and citation patterns. The analysis of research paper publications reveals a consistent upward trajectory over the years, characterized by periodic declines and subsequent surges. Noteworthy peaks in 2013 and 2010 are observed in Scopus and PubMed, respectively, followed by marked declines, particularly notable between 2021 and 2023. PubMed and Scopus data indicate that the United States and South Africa are the leading contributors. According to the PubMed and Scopus databases, the University of Cape Town and Stellenbosch University are the institutions that contribute the most.

Keyword and thematic analyses underscore the primary research focuses on drug-resistant tuberculosis (DR-TB), including drug combination therapy, microbiological analysis of sputum, therapeutic uses of antitubercular agents, drug resistance (DR), multidrug resistance (MDR), and *Mycobacterium tuberculosis*. The trend-topic analysis reveals dynamic shifts in research focus over time, transitioning from single-drug therapy to addressing drug resistance and highlighting the emerging need for effective drug therapy in cases of multidrug-resistant tuberculosis. Notably, most research papers on drug-resistant tuberculosis are single-country publications. Citation analysis in the Scopus database indicates that the average citation per year and mean total citation per year peaked during 2005-2006. This suggests a period of heightened impact and recognition within the research community during that timeframe. The study's findings may inform strategic planning for combating drug-resistant tuberculosis, ultimately contributing to future enhanced prevention, diagnosis, and treatment strategies.

## Introduction and background

Tuberculosis (TB) remains a persistent global public health challenge, with millions of new cases reported annually. The emergence and dissemination of drug-resistant strains of *Mycobacterium tuberculosis* further complicate control efforts, posing a substantial threat. Notably, the prevalence of drug-resistant TB (DR-TB) witnessed a 3% increase from 2020 to 2021, with 450,000 new cases of rifampicin-resistant TB (RR-TB) reported in the latter year. Regions in Eastern Europe and Central Asia, particularly Russia, showed alarming rates of multidrug-resistant TB (MDR-TB) in individuals previously treated [1]. Furthermore, global targets set at the first UN high-level meeting on TB for the 5 years 2018-2022 were not achieved. WHO estimated that 410,000 people had developed MDR or RR-TB globally in 2022 [2].

WHO uses five categories to classify cases of drug-resistant TB: isoniazid-resistant TB (INHR-TB), RR-TB, MDR-TB, extensively drug-resistant TB (XDR-TB), and pre-extensively drug-resistant TB. MDR-TB denotes resistance to isoniazid and rifampicin. Pre-extensively drug-resistant TB denotes resistance to rifampicin and any fluoroquinolone (a class of second-line anti-TB drugs). XDR-TB denotes resistance to rifampicin, plus any fluoroquinolones, and with the addition of at least one of either bedaquiline or linezolid [2,3]. Antitubercular drug resistance, encompassing both MDR-TB and XDR-TB, introduces complexities in treatment regimens and leads to treatment failures.

Compounding the challenge, the impact of the COVID-19 pandemic, as reported by WHO, continues to affect TB diagnosis and care adversely. This setback has even reversed the progress made in combating TB until 2019, evident in the 3.6% rise in the incidence rate of TB in 2021 compared to 2020. This marks a shift from the nearly 2% annual decrease observed over the past two decades [1,4]. However, the latest WHO report- “The Global Tuberculosis Report 2023,” mentioned that there was an encouraging recovery in the number of people being diagnosed with TB and treated in 2022, which has started to reverse or mitigate the damaging impact of the pandemic [2].

The WHO report highlighted persistent challenges, particularly in improving suboptimal TB treatment, enhancing prevention efforts, and expanding TB screening and diagnostic test services [5]. The latest report expanded its scope to incorporate strategies including TB prevention and care among children, universal access to diagnostics and improved treatment regimens, and primary healthcare integration, investment in public health infrastructure, and safeguarding TB services during emergencies. Emphasis is placed on enabling TB research and innovation, promoting access to affordable medicines, and ensuring multisectoral accountability through ambitious national TB strategic plans [2].

Despite being preventable and usually curable, TB remained the world’s second leading cause of death from a single infectious agent in 2022, after COVID-19, and caused almost twice as many deaths as HIV/AIDS [2]. Understanding the dynamics, trends, and key factors influencing antitubercular drug resistance can help devise informed strategies to combat this escalating problem. By analyzing patterns in scientific literature, collaboration networks, and research themes, bibliometric analysis can offer valuable insights into the state of current knowledge, identify research gaps, and guide future directions in the field.

This bibliometric study aims to systematically review and analyze the existing body of literature related to clinical trials on antitubercular drug resistance and identify global research trends, most relevant contributors, collaboration, scientific hotspots, and emerging themes that may shape the trajectory of antitubercular drug resistance research in the coming years.

## Review

Materials and methods

On February 5, 2024, an electronic search was conducted on the PubMed and Scopus databases to identify research papers addressing drug-resistant antituberculosis. The search employed the strategy -(Antitubercular) AND (Drug) AND (Resistance) AND (Tuberculosis). Only clinical trials published in English were included in the analysis. In PubMed, filters were applied for article type and language. In Scopus, filters for source, article type, and language were applied. The study selection process adhered to the PRISMA guidelines, and a flow chart was generated to illustrate this process [6].

The gathered data was exported to text files for subsequent analysis. Biblioshiny, a web-based app for comprehensive science mapping analysis, was utilized to identify the most relevant contributors, collaboration dynamics, author productivity analysis, citation analysis, and publishing trends analysis [7]. Network analysis and density visualization were done using VOSviewer version 1.6.20 [8]. VOSviewer is a software tool for constructing and visualizing bibliometric networks, helping users analyze and explore relationships among scientific publications, authors, and keywords. Microsoft Excel was also used for the graphical representation of the results. By analyzing publication trends, most relevant contributors, citation networks, and collaboration dynamics, we aim to shed light on the following areas.

Global Research Trends

We analyzed the number of publications worldwide to identify geographic regions actively engaged and track the evolution of research focus on DR-TB using the Biblioshiny App.

Scientific Hotspots

We identified the field's most productive institutions, sources, and countries to reveal centers of excellence using the Biblioshiny App.

Collaborations

A co-authorship analysis was done using VOSviewer software, while the collaboration frequency of most contributing countries was identified using the Biblioshiny App.

Emerging Themes and Future Directions

We analyzed the keywords, their cooccurrences, and themes associated with high-impact publications. We performed thematic and trend-topic analyses and generated a word cloud using the Biblioshiny App. For keyword cooccurrence analysis, we used VOSviewer software.

Results

**Figure 1:**
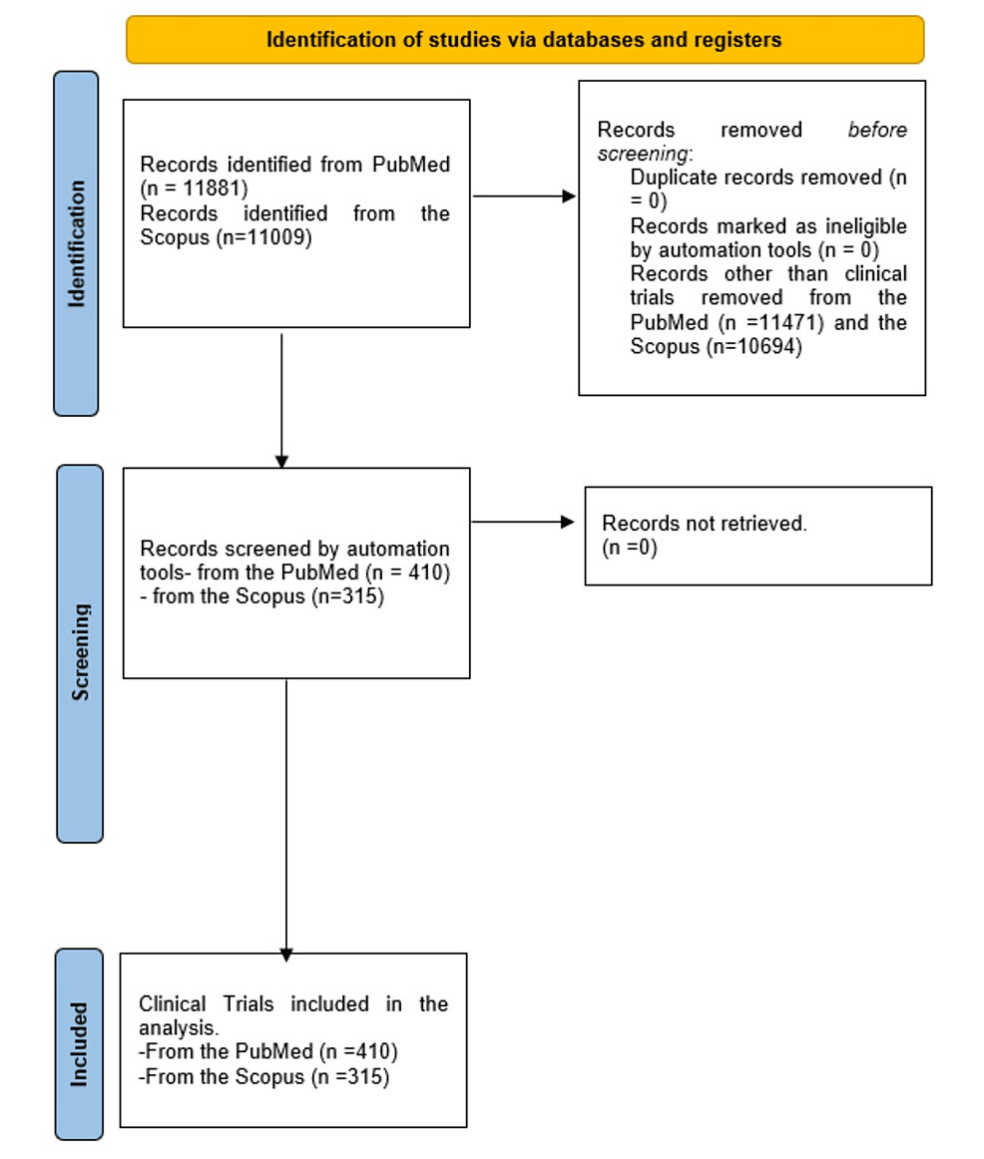
Flowchart of the process of selection of studies in PubMed and Scopus databases. Image Credit: Namrata Dagli

A total of 11,881 results appeared from an online search in the PubMed database, among which 410 were clinical trials, 175 were randomized clinical trials, 1746 were reviews, 183 were meta-analyses, and three were books and documents. All articles other than clinical trials were excluded from the analysis. A search in the Scopus database retrieved 11,009 documents. After applying filters for source, article type, and language, 7,609 articles published in English were obtained (Figure 1), and 315 articles were clinical trials. The selected articles were published between 1965 and 2024 in both databases.

Bibliometric analysis of data obtained from the PubMed and Scopus databases

Annual Scientific Production of Research Papers on Drug-Resistant Tuberculosis

**Figure 2:**
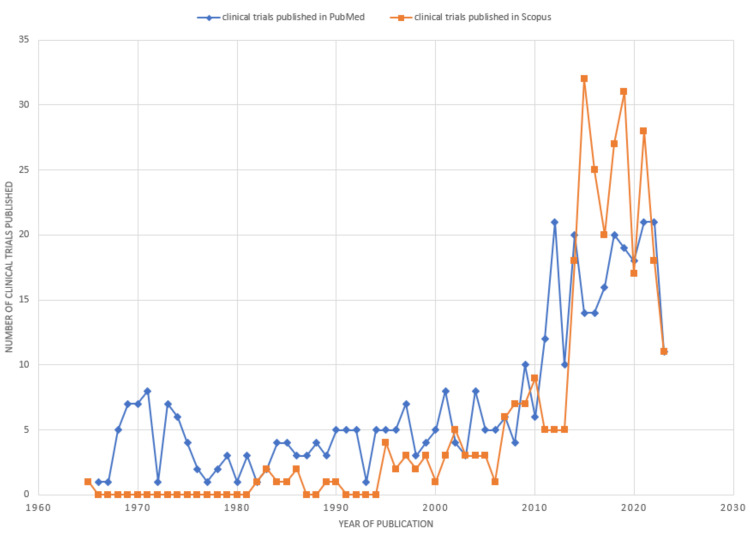
Annual scientific production of research papers on drug-resistant tuberculosis. Image Credit: Namrata Dagli

The graph (Figure 2) shows an irregular pattern in the quantity of research papers released over the years. The patterns in the graphs from both databases exhibit considerable similarity. Initially, no clinical trials were published in Scopus from 1966 to 1981, and the annual publications were significantly lower compared to PubMed until 2001. Subsequently, irregularities in publication patterns become evident in the graph. A significant surge was noticeable in 2013 in Scopus, indicating a noteworthy increase in published clinical trials on drug-resistant TB, followed by a spike in 2010 in PubMed. However, these notable peaks are followed by marked declines in the subsequent years. The most prominent decrease in the number of published clinical trials is observed between the years 2021 and 2023.

Co-Authorship Analysis of Authors

**Figure 3:**
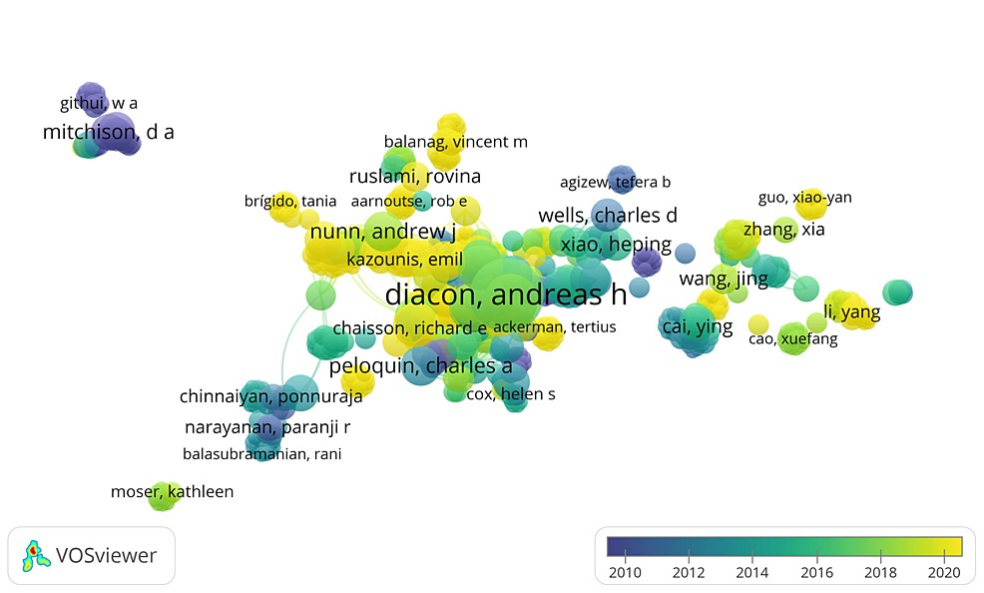
Overlay visualization of co-authorship analysis of authors based on PubMed data (Weight: Documents, Scores: Average Publications). Note: The size of the nodes indicates the number of publications. Image Credit: Namrata Dagli

In the PubMed database, we identified 2,438 authors who have published at least one paper on drug-resistant TB. Setting thresholds at two, three, four, and five documents, the corresponding numbers of authors identified were 419, 138, 62, and 33, respectively. We selected the top 1,000 authors based on Total Link Strength (TLS), resulting in 771 connected authors. The authors with the highest TLS are represented in Figure 3. This network comprised 26 clusters, with author counts ranging from 5 to 53, with 8240 links and 9596 TLS. The analysis reveals that author AH Diacon has published 24 clinical trials with the highest TLS, 276, in the PubMed database.

**Figure 4:**
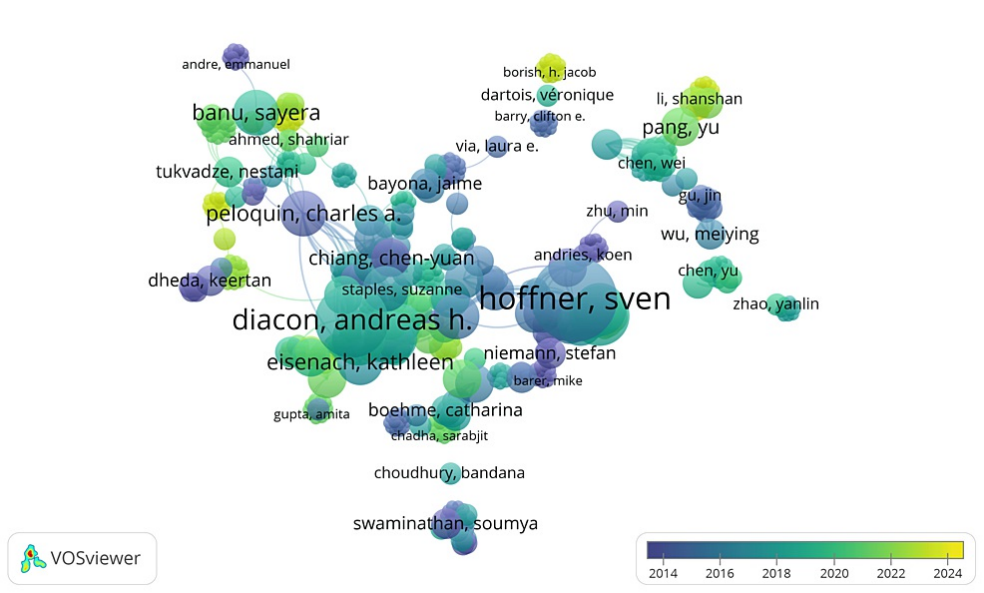
Overlay visualization of co-authorship analysis of authors based on Scopus data. (Weight: Documents, Scores: Average Publications). Note: The size of the nodes indicates the number of publications. Image Credit: Namrata Dagli

In the Scopus database, 2,244 authors were identified with at least one publication on drug-resistant TB. Setting thresholds of two, three, four, and five documents, the corresponding number of authors counts were 281, 73, 33, and 12. We calculated the TLS of co-authorship links for these 2,244 authors and selected the top 1,000 based on TLS. Six hundred twenty-four authors were connected and organized into 23 clusters, with author counts ranging from 5 to 53. This network comprised 6,031 links and 6,672 TLS. The graph in Figure 4 includes the authors with the highest TLS among 624 connected authors. The analysis reveals that AH Diacon has published nine clinical trials with 947 citations and the highest TLS value of 104. At the same time, author Sven Hoffner has the highest number of citations, 2213 in 11 clinical trials, and a TLS value of 88. The yellow area in both graphs indicates the most recent data.

Author's Productivity Analysis

**Figure 5:**
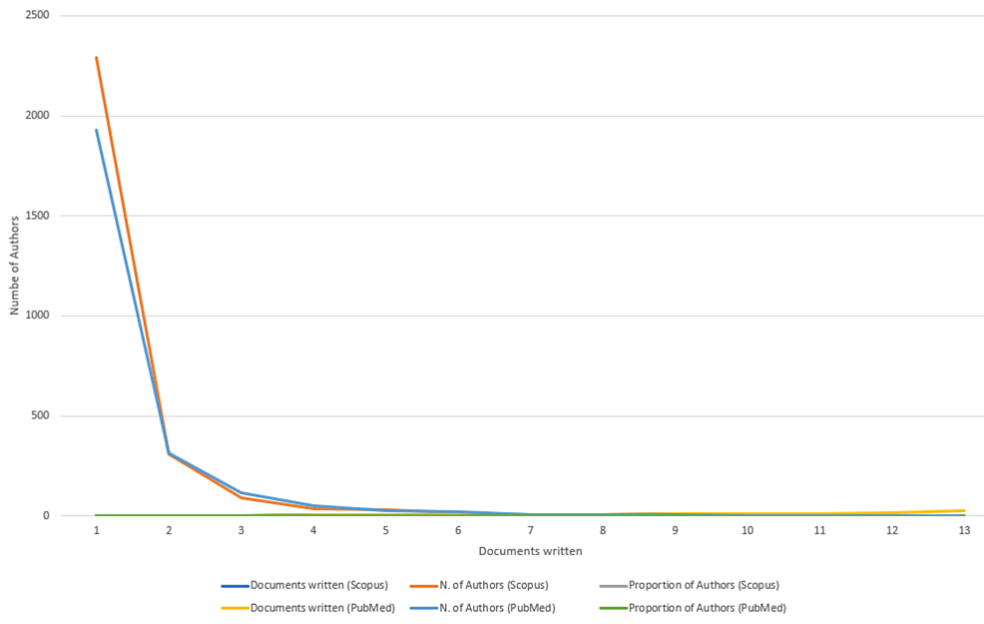
Author’s productivity analysis through Lotka’s Law. Image Credit: Namrata Dagli

Figure 5 depicts the authors' productivity in PubMed and Scopus databases. The graph clearly illustrates that the overall productivity of authors is relatively low, with the majority having authored fewer than two documents. The analysis highlights that only six authors have surpassed the threshold of writing more than 10 research papers in both databases.

Most Contributing Countries, Sources, and Institutions

**Figure 6:**
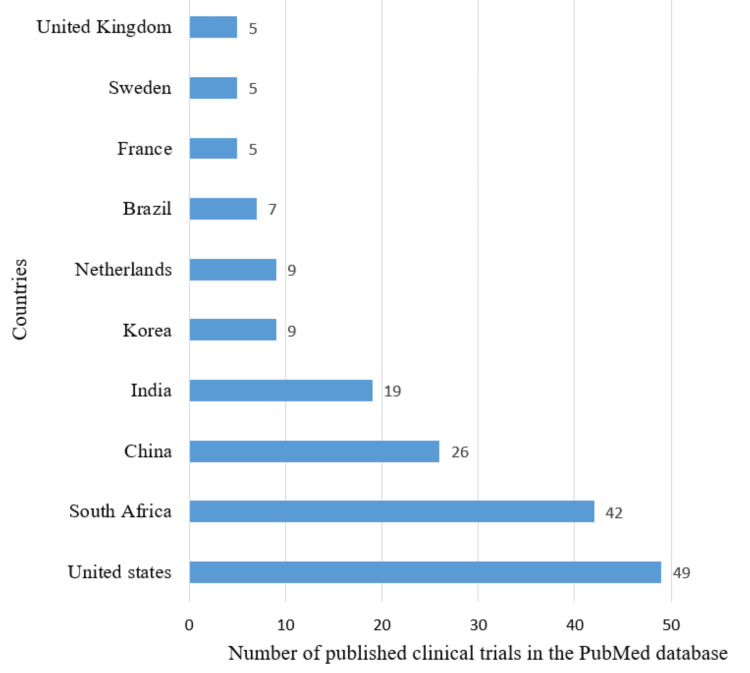
Most contributing countries in research papers related to drug-resistant tuberculosis based on the PubMed database. Image Credit: Namrata Dagli

**Figure 7:**
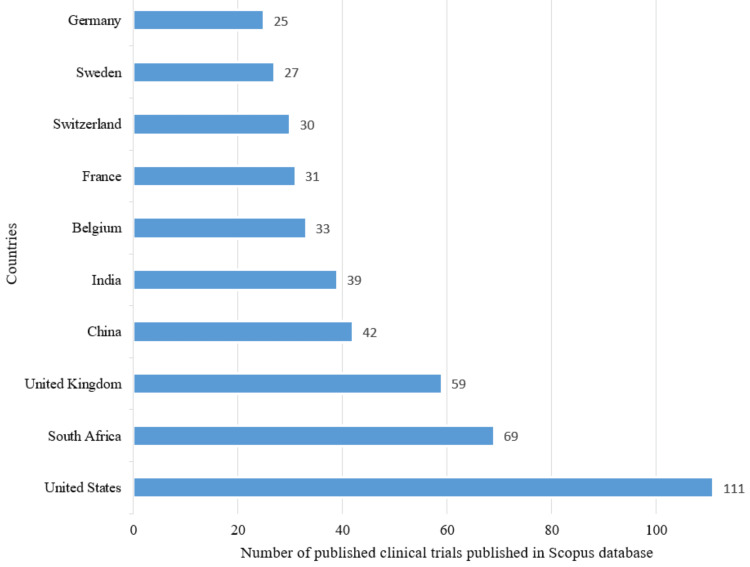
Most contributing countries in research papers related to drug-resistant tuberculosis based on the Scopus database. Image Credit: Namrata Dagli

The leading contributors, based on data from both PubMed and Scopus, are the United States and South Africa. According to both databases, other countries making significant contributions include the United Kingdom, China, India, France, and Sweden. Figure 6 denotes the PubMed database, and Figure 7 illustrates the Scopus database. Notably, the United States played a prominent role, accounting for 111 (35.2%) clinical trials out of the 315 published in Scopus and 49 (11.95%) clinical trials out of the 410 published in PubMed.

**Figure 8:**
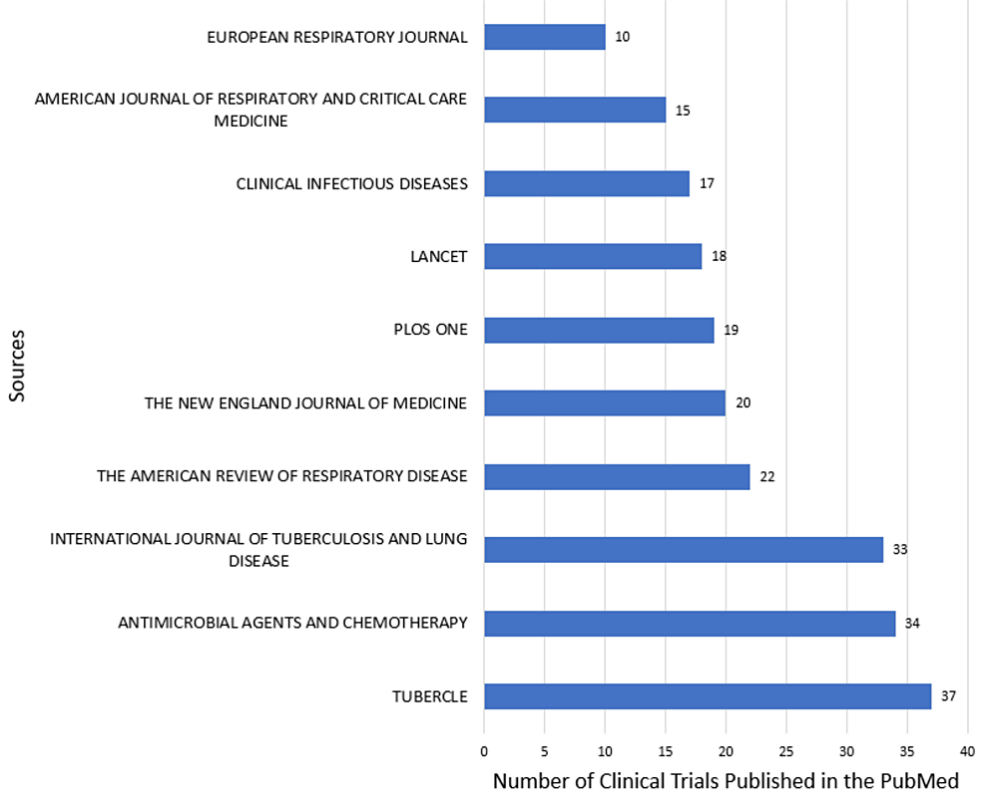
Most relevant sources in research papers related to drug-resistant tuberculosis in the PubMed database. Image Credit: Namrata Dagli

**Figure 9:**
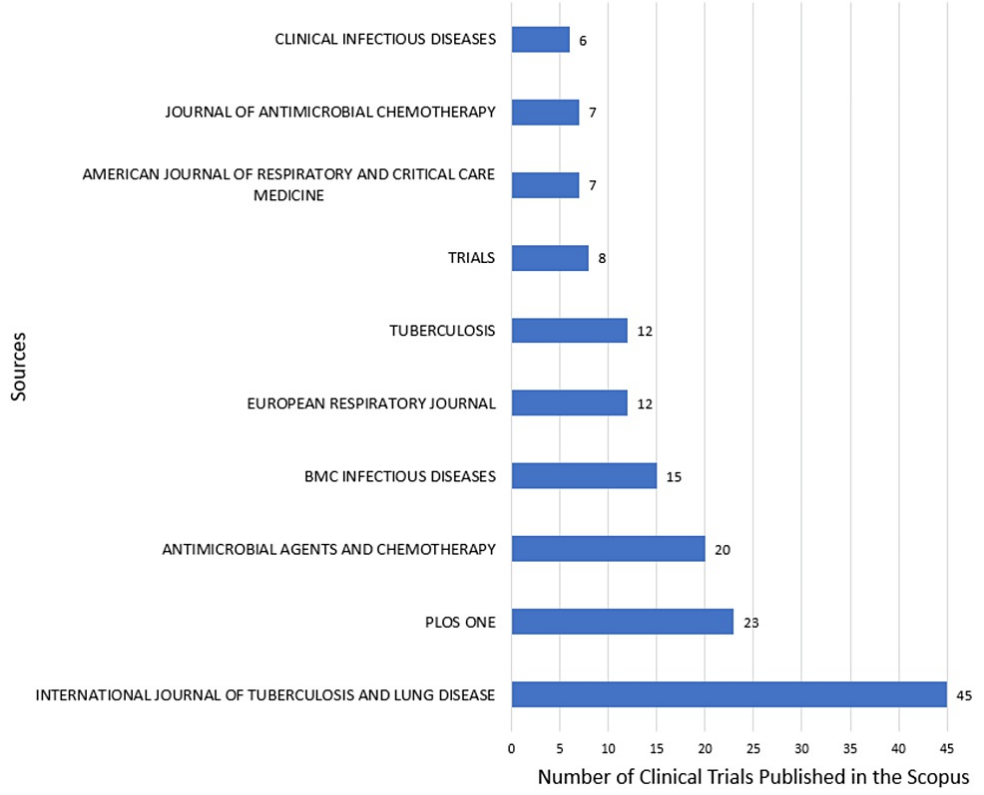
Most relevant Sources in Research papers related to drug-resistant tuberculosis in the Scopus database. Image Credit: Namrata Dagli

The 10 most productive sources are the same in both databases. The most productive sources are the International Journal of Tuberculosis and Lung Disease, with 45, and the Tubercle, with 37 clinical trials published in PubMed (Figure 8) and Scopus (Figure 9) databases, respectively.

**Figure 10:**
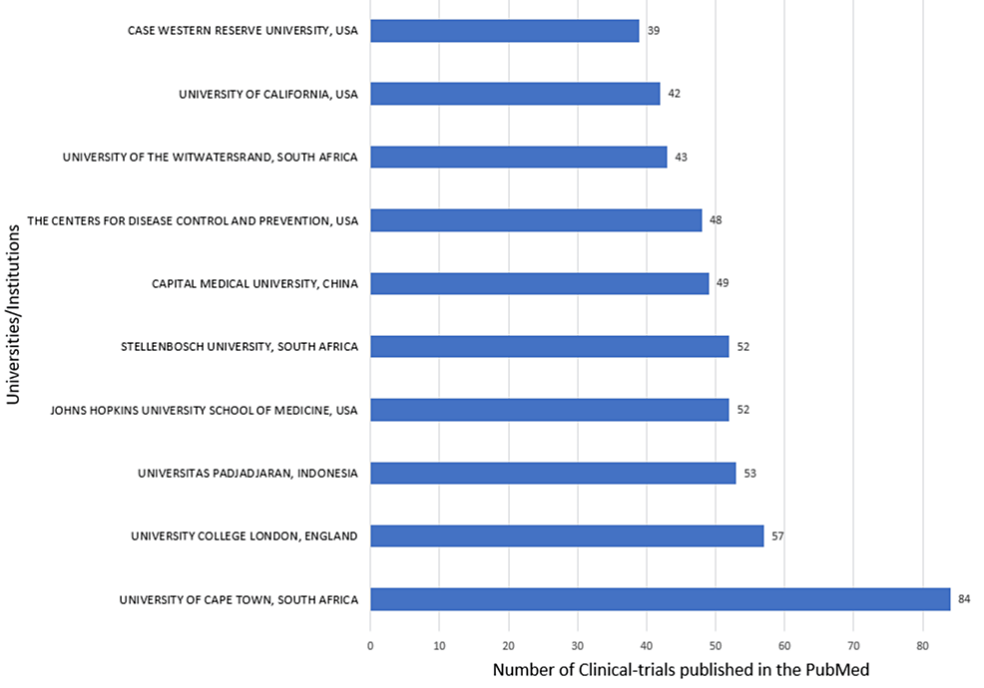
Most relevant universities/institutions based on PubMed database. Image Credit: Namrata Dagli

**Figure 11:**
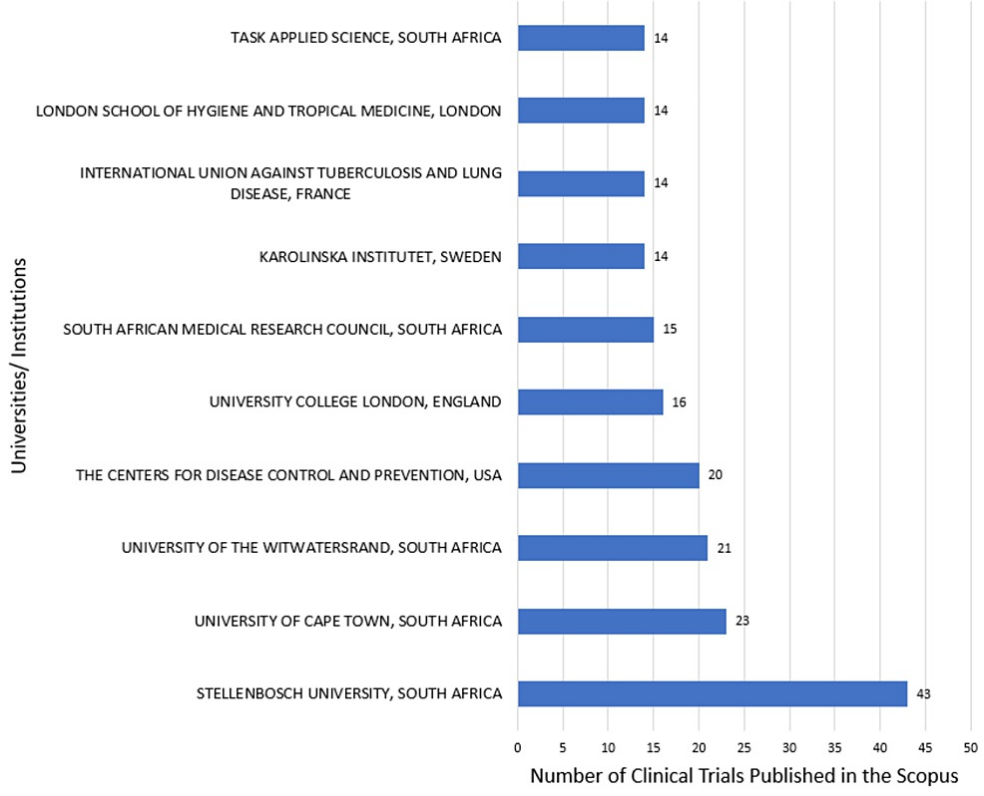
Most relevant universities/institutions based on Scopus. Image Credit: Namrata Dagli

The most relevant institutions in both databases are Stellenbosch University, University of Cape Town, University of the Witwatersrand, the Centers for Disease Control and Prevention, and University College London. According to the PubMed and Scopus databases, the institutions that contribute the most are the University of Cape Town and Stellenbosch University PubMed (Figure 10) and Scopus (Figure 11), respectively.

Analysis in PubMed database

Keyword Analysis

**Figure 12:**
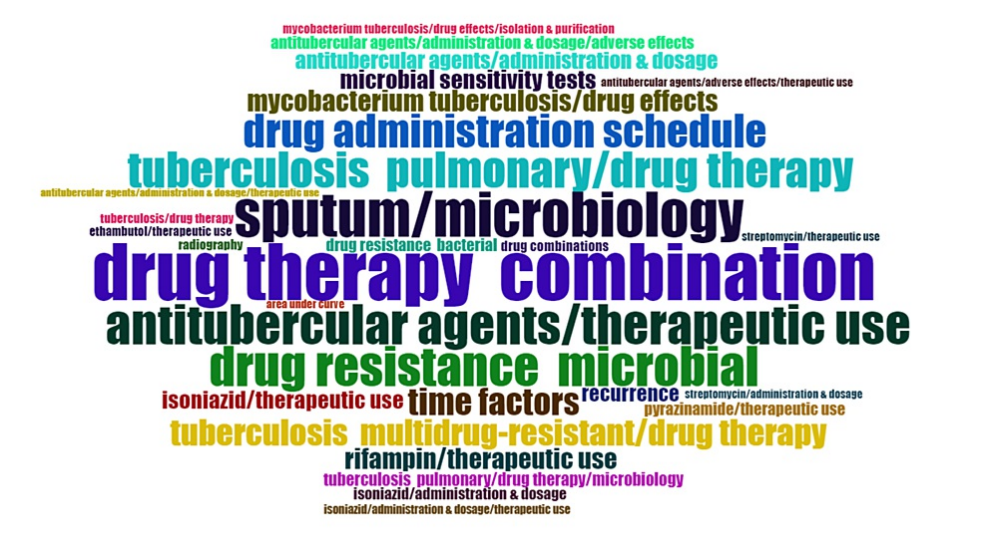
Word Cloud, a visual representation of the most frequently occurring words. The size of each word is proportional to its frequency in the text. Image Credit: Namrata Dagli

Word clouds are beneficial for visually summarizing large volumes of text and identifying patterns or recurring themes across a corpus of documents. This helps researchers gain insights into specific field subject areas. Typically, the more frequently a word appears in the documents, the larger it seems in the word cloud. Figure 12 highlights the most often used 30 keywords. The following keywords, nonspecific to drug-resistant TB, were removed from the analysis - human, humans, infant, adult, middle-aged, animals, aged, young adult, child preschool, aged 80 and over, child preschool, infant newborn, random allocation, and clinical trials as a topic. Figure 12 indicates that research on drug-resistant TB focused on drug combination therapy, microbiological analysis of sputum, therapeutic uses of antitubercular agents, drug resistance, and multidrug resistance. It also highlights that studies mainly were centered on therapeutic uses of streptomycin, rifampin, isoniazid, pyrazinamide, and ethambutol.

Keyword Cooccurrence Analysis

**Figure 13:**
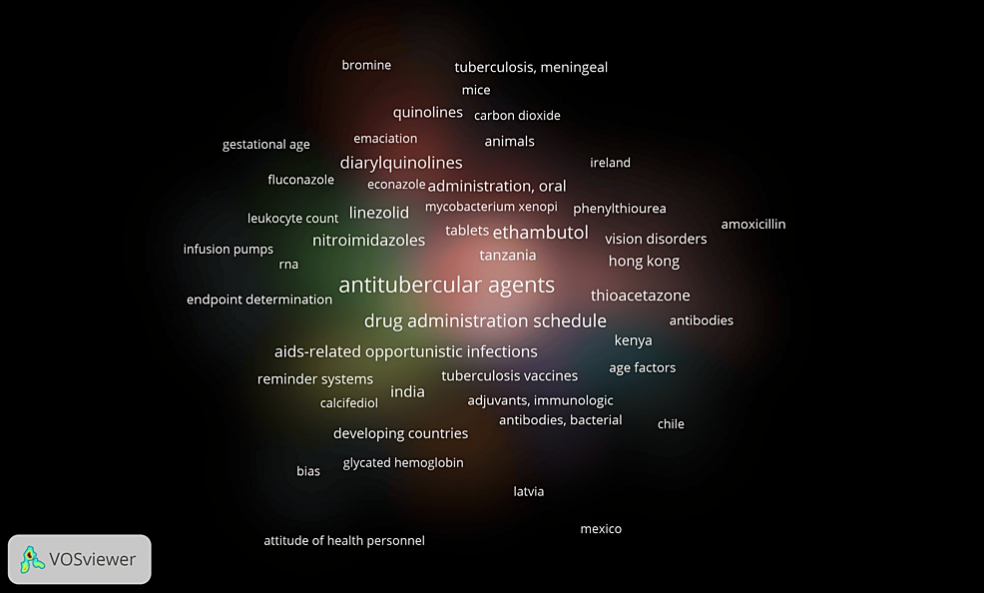
Cluster density visualization of cooccurrence of MeSH keywords (Weight: occurrences). Image Credit: Namrata Dagli

Cluster density visualization in bibliometric analysis is a graphical representation that illustrates the density and distribution of clusters or groups of related terms or documents. The study identified 700 Medical Subject Headings (MeSH) keywords. Setting frequency thresholds of 2, 3, 4, and 5, the corresponding number of keyword counts were 316, 226, 167, and 132. For each of the 700 keywords, cooccurrence links with other keywords, and TLS were calculated using VOSviewer software. We removed 32 nonspecific keywords, and the analysis included 668 items connected with 9,388 links and 20,665 TLS. Thirty-six clusters were identified, with items ranging from 1 to 57. Figure 13 represents the density visualization of MeSH keywords with the highest TLS values used across the research papers on drug-resistant TB. The analysis revealed that the research was mainly focused on the administration schedule of antitubercular agents. The other keywords identified are India, Latvia, Chile, Kenya, Ireland, and Hong Kong, which indicate the regions where the research has been conducted more frequently. This also shows that these regions might have a high prevalence of TB or DR-TB.

Trend Topic Analysis and Thematic Analysis

**Figure 14:**
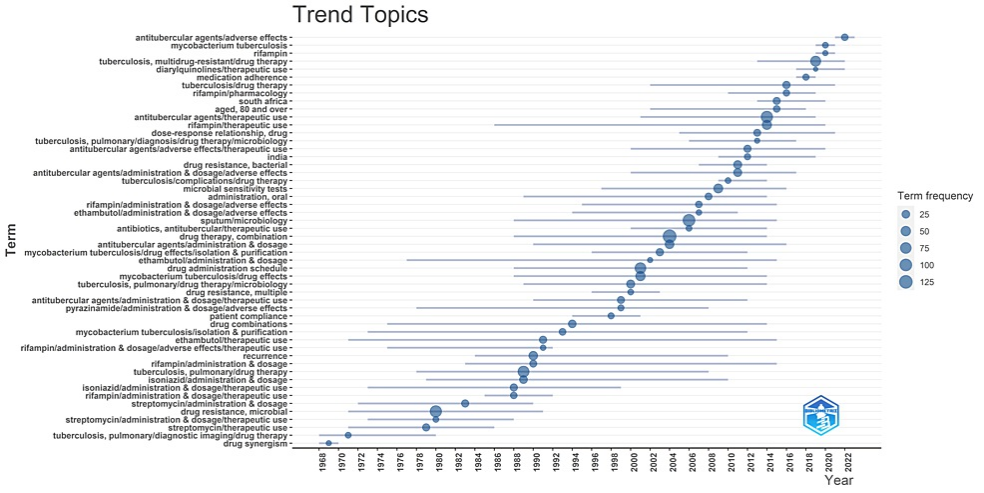
Trend-topic analysis of research papers on drug-resistant tuberculosis. (Field- Keywords plus, Minimum frequency of words -7, maximum two terms per year). Image Credit: Namrata Dagli

In the research conducted between 1968 and 1987, the primary focus was on pulmonary TB and its diagnostic imaging and the administration, dosage, and therapeutic use of streptomycin. Additionally, investigations centered around drug synergism during this period. Between 1988 and 2005, the research emphasized the dosage, administration, and therapeutic use of rifampin, isoniazid, ethambutol, and pyrazinamide. Researchers also explored the adverse effects of rifampin and pyrazinamide, drug combinations, patient compliance, recurrence, multiple drug resistance, and the isolation and purification of *M. tuberculosis*. After 2005, the main areas of investigation included sputum microbiology, microbial sensitivity tests, and adverse effects of rifampin, ethambutol, and other antitubercular agents. The focus extended to oral administration, bacterial drug resistance, and drug therapy for MDR-TB. This analysis highlights the dynamic shifts in research focus over time, transitioning from single-drug therapy to addressing drug resistance and the emerging need for effective drug therapy in cases of MDR-TB (Figure 14).

Thematic Analysis

**Figure 15:**
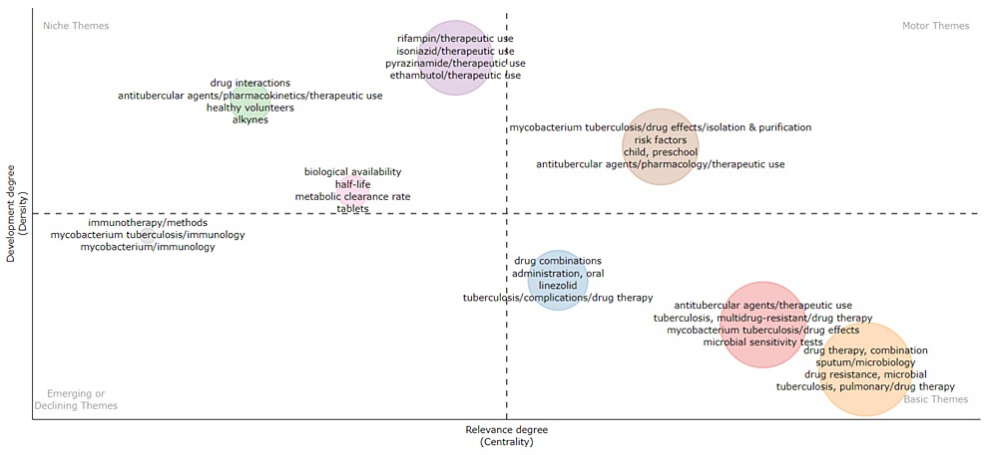
Thematic map of research themes on drug-resistant tuberculosis. Image Credit: Namrata Dagli

The thematic map visually represents the relationship between the most frequently used keywords in published clinical trials on DR-TB. The larger circle size represents more frequency of occurrence of the keywords. It is divided into four categories: motor themes, basic themes, niche themes, and emerging or declining themes. Basic themes, firmly established within the field, include three clusters, encompassing the terms related to pulmonary TB, drug resistance, drug combination therapy, and microbial sensitivity test. Motor themes represent the most relevant and developed themes, including - isolation, purification of *M. tuberculosis*, drug effects on *M. tuberculosis*, therapeutic use and pharmacology of antitubercular agents, and risk factors. Niche themes include the highly developed themes but are of limited importance- the therapeutic use of first-line drugs for TB and antitubercular agents, drug interaction, bioavailability, half-life, and metabolic clearance rate of antitubercular drugs. Emerging and declining themes include weekly developed and marginal themes - *M. tuberculosis* and immunology (Figure 15).

**Figure 16:**
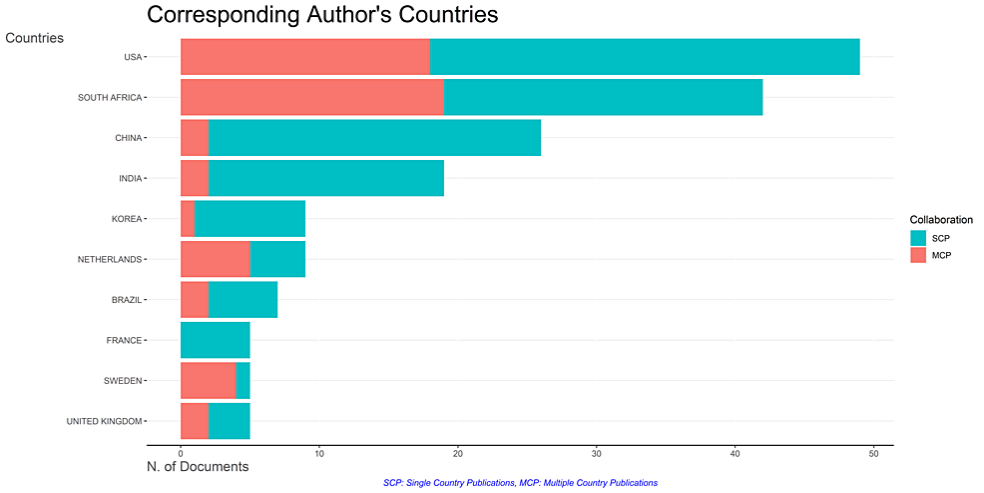
Analysis of the most relevant countries and their collaboration frequency in publishing clinical trials on drug-resistant tuberculosis. Image Credit: Namrata Dagli

**Figure 17:**
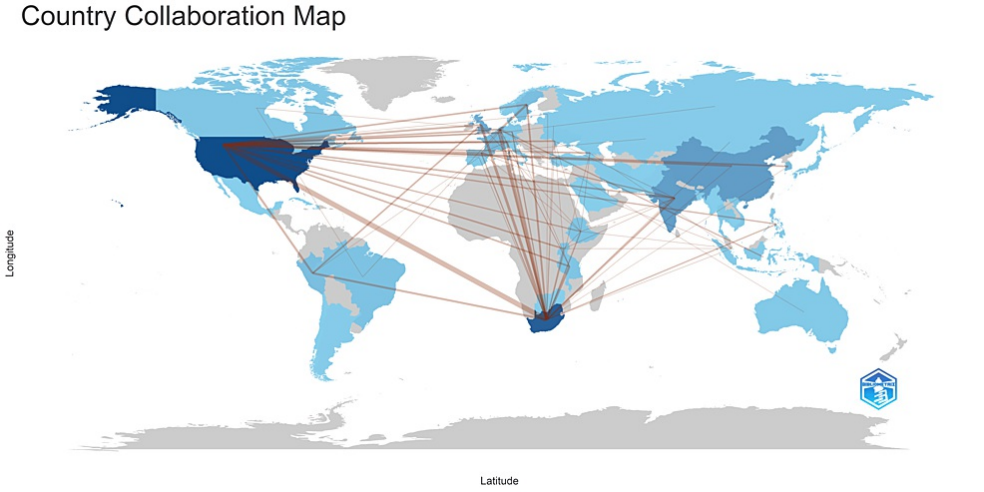
Country collaboration map. Note: The degree of color represents the frequency of collaboration. Image Credit: Namrata Dagli

Figure 16 illustrates that most clinical trials are published as single-country publications (SCP). South Africa exhibits the highest frequency of collaboration, followed by the USA. France did not engage in any collaborations with other countries. China, India, Brazil, and the United Kingdom demonstrate comparable collaboration frequencies. Additionally, Figure 17 depicts the collaboration frequencies of countries in publishing clinical trials on drug-resistant TB.

Citation analysis of the data obtained from the Scopus database

Most Globally Cited Clinical Trials on Drug-Resistant Tuberculosis

**Figure 18:**
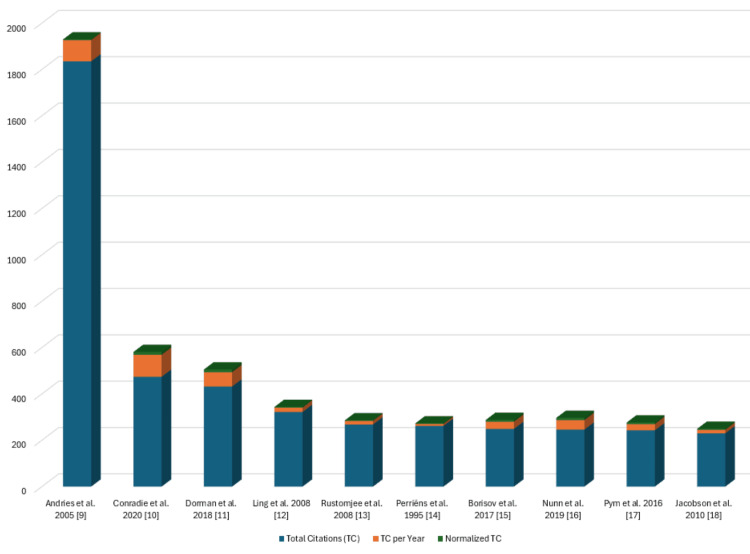
Most globally cited clinical trials on drug-resistant tuberculosis. TC: total citations Image Credit: Namrata Dagli

**Figure 19:**
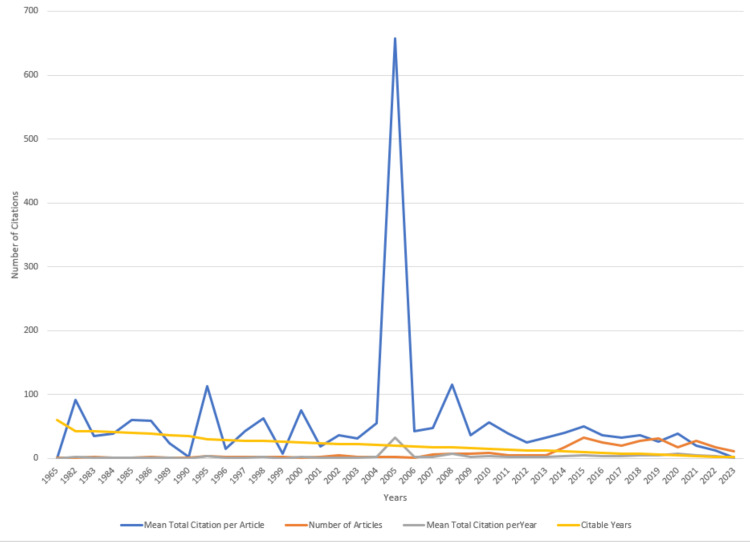
Average citations per year in clinical trials on drug-resistant tuberculosis. Image Credit: Namrata Dagli

Figure 18 displays the clinical trials on drug-resistant TB that have garnered the highest global citations [9-18]. The clinical trial published by Andries et al. in 2005 stands out with the highest total citations, reaching 1835 and 91.75 per year [9]. Additionally, the clinical trial by Conradie et al. in 2020 achieved the peak value for total citations per year at 94.8 [10]. Figure 19 illustrates a distinct irregularity in the citation pattern. The average citation per year and mean total citation per year are the maximum in 2005-2006. There is a remarkable increase in the number of published clinical trials after 2013, but due to fewer citable years, the increase in citations is not significant (Figure 19).

Discussion

The Annual Scientific Production of Research Papers on drug-resistant TB exhibits an irregular pattern with significant peaks in 2013 and 2010 in Scopus and PubMed, respectively, followed by marked declines, particularly notable between 2021 and 2023. The decline might be due to the post-pandemic impact on research activities worldwide. Many researchers and institutions shifted their attention and resources towards understanding and addressing the challenges posed by the pandemic, potentially leading to a decrease in publications on other topics, including DR-TB. We analyzed two databases in this study to enhance the robustness and depth of bibliometric analyses. The author's productivity analysis through Lotka's law revealed that the overall productivity of authors is relatively low, with the majority having authored fewer than two documents. According to the PubMed and Scopus databases, the institutions that contributed the most are the University of Cape Town and Stellenbosch University.

The word cloud generated by the Biblioshiny App highlights that research on drug-resistant TB focused on drug combination therapy, microbiological analysis of sputum, therapeutic uses of antitubercular agents, drug resistance, and multidrug resistance. The trend-topic analysis highlights the dynamic shifts in research focus over time, transitioning from single-drug therapy to addressing drug resistance and the emerging need for effective drug therapy in cases of multidrug-resistant TB. Thematic analysis suggests that the most relevant and developed themes are isolation, purification of *M. tuberculosis*, drug effects on *M. tuberculosis*, therapeutic use and pharmacology of antitubercular agents, and risk factors. Most research papers on drug-resistant TB are SCPs. The citation analysis in the Scopus database revealed that the average citation per year and mean total citation per year were the maximum in 2005-2006.

The findings of the bibliometric study by Mardaneh et al. indicate a recent rise in scientific publications addressing drug-resistant TB, primarily comprised articles and reviews. Most of these publications originate from the USA, India, and South Africa. Additionally, the study highlights that subjects such as drug resistance, care, treatment, drug activity, patient, and drug dose therapy regimen have the highest publication rates on drug-resistant TB [19]. The findings are like our study. The leading contributors, based on data from both PubMed and Scopus, are the United States and South Africa. Additionally, the cooccurrence analysis of MeSH keywords in our study also suggests that the focus of clinical trials on drug-resistant TB was on the administration schedule of antitubercular agents.

Another bibliometric study of 100 most cited articles on TB published in the Web of Sciences identified the New England Journal of Medicine as the most relevant source and the University of Cape Town as the leading institution. These results are like our study but differ due to the data from different databases. Our analysis in PubMed also identified the University of Cape Town as the leading institution and the New England Journal of Medicine as the fifth most relevant source [20].

Another bibliometric analysis identified the foremost journal in this field, the International Journal of Tuberculosis and Lung Disease. The Centers for Disease Control and Prevention emerged as the primary contributing institution. The countries with the highest productivity included the USA, India, the UK, South Africa, and China. Most collaborations occurred between the USA, the UK, and South Africa [21]. The International Journal of Tuberculosis and Lung Disease is the relevant journal for our study in Scopus database analysis. PubMed analysis also identified it as one of the 10 most relevant journals, and the Centers for Disease Control and Prevention is recognized as one of the 10 most relevant institutions by both PubMed and Scopus in our analysis. The findings related to the most productive countries and their collaborations are like ours.

Another bibliometric analysis of the top 50 cited articles published between 1982 and 2014 identified that first-line drugs such as isoniazid and rifampicin were the most studied. The word cloud in our study also highlighted that studies were mainly centered on therapeutic uses of streptomycin, rifampin, isoniazid, pyrazinamide, and ethambutol. The two most relevant journals identified were the New England Journal of Medicine, which published the most literature, and the American Journal of Respiratory and Critical Care Medicine. Our analysis identified them as one of the 10 most relevant journals. The most productive country identified is the same as determined by our study [22].

A bibliometric analysis by Merdan and Etiz indicated that countries with the highest estimated numbers of TB cases do not make considerable scientific contributions to TB research. This is also observed in our study [23]. The countries that have made the most significant contributions to published clinical trials are the USA and South Africa. In contrast, the three nations accounting for 42% of the estimated global number of individuals developing multidrug-resistant TB in 2022 were India, the Philippines, and the Russian Federation. However, four countries listed by the WHO as '30 high multidrug-resistant TB burden countries - India, China, South Africa, and Korea - are identified as the leading contributors in terms of the number of published papers in our analysis. Notably, the resurgence in incidence cases in 2021 and 2022 is likely attributed to individuals whose diagnosis was delayed due to disruptions caused by COVID-related factors. Many bibliometric analyses have already been published, but our analysis included both PubMed and Scopus databases, which enabled us to analyze the literature more comprehensively. In addition to identifying leading contributors, we have performed thematic, citation, and trend-topic analyses. Through this bibliometric analysis, we aspire to contribute to the ongoing efforts to address the challenges of drug-resistant TB. Our study employed automated tools to screen articles, mitigating subjective bias and enabling large-scale data analysis.

Despite its advantages, the study had its limitations. Firstly, it is impossible to merge the data from the two databases, so the data from these databases was analyzed separately. Secondly, exploring each paper individually and checking their relevancy was not feasible given the many articles included. Detailed analysis of drug resistance development, mechanism, and novel treatment approaches requires in-depth systematic reviews. Thirdly, citation analysis only included Scopus data as this analysis is not possible with data from the PubMed database, and thematic and keyword analysis only included the PubMed database as none of the software used supported these analyses with data from the Scopus database. Another major limitation of the VOSviewer and Biblioshiny App is their dependency on bibliographic data quality and completeness, as inaccuracies or missing information in the underlying dataset can impact the accuracy and reliability of the generated bibliometric maps and analyses. Nevertheless, this study serves as a valuable resource for identifying research trends, leading researchers, countries, and organizations, and offering valuable insights for new researchers on drug-resistant TB.

Future recommendations for research on DR-TB include the development of novel therapies, precision medicine approaches, and vaccine development. Strengthening surveillance, improved diagnostics, and patient-centered interventions are needed to enhance adherence. Research should also focus on health systems strengthening, global collaboration, and antimicrobial stewardship. Additionally, community engagement and education programs are crucial to raising awareness and reducing stigma around DR-TB. These recommendations might help address the evolving challenges and improve the management of DR-TB.

## Conclusions

The Annual Scientific Production of Research Papers on drug-resistant TB exhibits an irregular pattern with significant peaks in 2013 and 2010 in Scopus and PubMed, respectively, followed by marked declines, particularly notable between 2021 and 2023. The authors’ productivity analysis through Lotka’s Law revealed that the overall productivity of authors is relatively low, with the majority having authored fewer than two documents. The leading contributors, based on data from both PubMed and Scopus, are the United States and South Africa. According to the PubMed and Scopus databases, the institutions that contributed the most are the University of Cape Town and Stellenbosch University. The word cloud highlights that research on drug-resistant TB focused on drug combination therapy, microbiological analysis of sputum, therapeutic uses of antitubercular agents, DR, and MDR. It also highlights that studies were mainly centered on therapeutic uses of first-line therapy for TB. The cooccurrence analysis of keywords also suggests that the focus of clinical trials on DR-TB was on the administration schedule of antitubercular agents. This trend-topic analysis highlights the dynamic shifts in research focus over time, transitioning from single-drug therapy to addressing drug resistance and the emerging need for effective drug therapy in cases of MDR-TB. Thematic analysis suggests that the most relevant and developed themes are isolation, purification of *M. tuberculosis*, drug effects on *M. tuberculosis*, therapeutic use and pharmacology of antitubercular agents, and risk factors. Most research papers on drug-resistant TB are SCPs. South Africa exhibits the highest frequency of collaboration, followed by the USA. The citation analysis in the Scopus database revealed that the average citation per year and mean total citation per year were the maximum in 2005-2006. By synthesizing and visualizing the current literature, this study aims to empower researchers, policymakers, and healthcare professionals with a comprehensive understanding of the current landscape, ultimately fostering collaborative initiatives and accelerating global efforts to overcome this critical challenge and achieve the goal of eliminating TB as a public health threat.
